# Efgartigimod-associated Kaposi’s varicelliform eruption and herpetic conjunctivitis in a patient with seropositive ocular myasthenia gravis: a case report and review

**DOI:** 10.3389/fimmu.2024.1409480

**Published:** 2024-08-01

**Authors:** Lingzhi Ge, Yanyan Li, Ying Sun, Wenfang Chen, Xiaoli Ni, Fangli Wei, Zhen Mu

**Affiliations:** ^1^ Department of Dermatology, The Second Affiliated Hospital of Shandong First Medical University, Tai’an, China; ^2^ Department of Graduate Studies, Shandong First Medical University & Shandong Academy of Medical Sciences, Jinan, China

**Keywords:** neonatal Fc receptor antagonist, efgartigimod, herpes simplex virus, Kaposi’s varicelliform eruption, herpetic conjunctivitis, myasthenia gravis

## Abstract

**Background:**

Efgartigimod (Efgartigimod alpha fcab, Vyvgart™) is a pioneering neonatal Fc receptor (FcRn) antagonist for the treatment of severe autoimmune diseases mediated by pathogenic immunoglobulin G (IgG) autoantibodies, including myasthenia gravis (MG). It is a well-tolerated drug with minor side effects, such as headache and upper respiratory (lung) and urinary tract infections. Here, we present a case of Kaposi’s varicelliform eruption (KVE) and herpetic conjunctivitis related to efgartigimod in a 60-year-old patient with ocular MG (OMG).

**Case description:**

A 60-year-old Chinese male suffered from acetylcholine receptor antibody positive (AChR Ab+) OMG for 8 years. During this period, he underwent first-line treatment with systemic corticosteroids, cyclosporine, cyclophosphamide, and so on, but had poor symptom improvement. On the recommendation of his attending neurologist, he received one cycle of intravenous efgartigimod (10mg/kg, once weekly for 4 weeks). The patient experienced fever, widespread painful blisters, and edema on the face on the third day after his last intravenous infusion. The patient also complained of increased secretions and a foreign body sensation in both eyes. Laboratory tests confirmed infection with herpes simplex virus (HSV). A diagnosis of efgartigimod-associated KVE and herpetic conjunctivitis was made. After intravenous administration (5mg/kg, 3 times a day, every 8 hours) for 10 days, the patient was cured without residual complications.

**Conclusions:**

This case is the first report of a patient with KVE and herpetic conjunctivitis related to efgartigimod in PubMed. This is rare and unusual. Clinicians should be alert to the rare symptoms related to efgartigimod.

## Introduction

Myasthenia gravis (MG) is a chronic autoimmune neuromuscular disorder that causes localized or general voluntary muscle weakness (1). Targeted immunotherapy has become a promising approach for treating MG, overcoming some limitations of traditional therapeutic approaches such as corticosteroids and nonsteroidal immunosuppressive therapy. Efgartigimod (Efgartigimod alpha fcab, Vyvgart™) is a first-in-class neonatal Fc receptor (FcRn) antagonist developed by argenx, which can cause IgG catabolism, leading to a decrease in overall IgG and pathological autoantibody levels (2). Some clinical studies are also investigating the effectiveness of efgartigimod for other autoimmune diseases, including bullous pemphigoid, chronic inflammatory demyelinating polyneuropathy, immune thrombocytopenia, autoimmune myositis, and pemphigus. Efgartigimod has a favorable safety profile with minor side effects such as headache and upper respiratory (lung) and urinary tract infections (3). There are concerns about the potential for an increased risk of infections in patients receiving FcRn antagonists due to decreased IgG levels. It is not recommended to receive live-attenuated or live vaccines during treatment (4).

Kaposi varicelliform eruption (KVE), also known as eczema herpeticum, is a rare and potentially life-threatening infection mainly caused by herpes simplex virus (HSV). Here, we describe a case of efgartigimod-associated KVE and herpetic conjunctivitis in a patient with ocular MG (OMG). To the best of our knowledge, this is the first report of a patient with KVE and herpetic conjunctivitis related to efgartigimod in PubMed. The main reason for reporting the case study is that KVE and herpetic conjunctivitis may be related to efgartigimod. This is rare and unusual. Clinicians should be alert to efgartigimod-associated KVE and herpetic conjunctivitis.

## Case description

### Patient presentation

**Figure 1:**
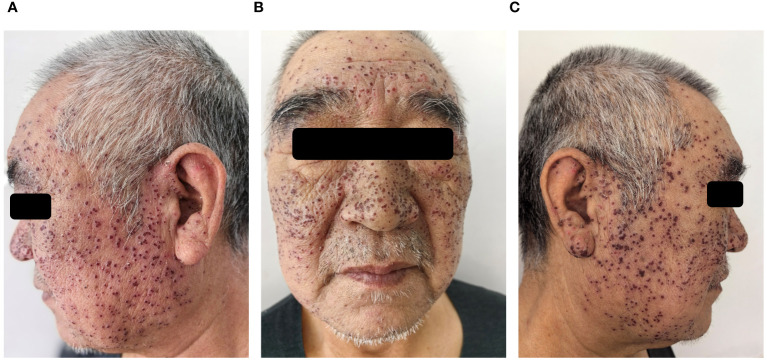
After four days of antiviral treatment with acyclovir, scabs formation at the site of vesicles and facial edema subsided **(A-C)**.

A 60-year-old Chinese male with acetylcholine receptor antibody positive (AChR Ab+) OMG presented to our dermatology clinic with a fever (38.5°C), widespread painful blisters, and edema on the face. The patient also complained of increased secretions and a foreign body sensation in both eyes. At the time of presentation, he had already finished one treatment cycle of intravenous efgartigimod (10mg/kg, once weekly for 4 weeks) for AChR Ab+ OMG. The rash appeared on the third day after his last intravenous therapy. The patient had suffered with AChR Ab+ OMG for 8 years and previous first-line treatment with systemic corticosteroids, cyclosporine, and cyclophosphamide showed a poor response. The patient had a history of mild seborrheic dermatitis on the face for more than 10 years and had received intermittent topical medication treatment in the past, but no topical hormone, tacrolimus, or other medication treatment for 3 years. The patient had no familial genetic predisposition or other infectious diseases. Dermatologic examination showed that the blisters were scattered on the edematous face and both auriculae (**Figure 1**). His pre- and infra-auricular lymph nodes were enlarged. Upon eye examination by an ophthalmologist, the discharge from both eyes was found to be watery mucus, the conjunctival was hyperemia and edema, and the cornea was clear with negative corneal fluorescein staining. 

### Laboratory findings

The cytological findings of Tzanck smears of a sample collected from the vesicles was ballooning multinucleated giant cells and eosinophilic inclusion bodies. A polymerase chain reaction (PCR) of the conjunctival specimen was positive for HSV-DNA. Laboratory investigations revealed elevations of lymphocyte% (LYMPH%), monocyte% (MONO%), and C-reactive protein (CRP) (**Table 1**). Additional laboratory analysis revealed normal liver enzyme level, normal creatinine level, normal blood glucose level, negative HIV and syphilis antibodies, and normal urine and stool analysis. An EKG and chest X-ray indicated no cardiac, pulmonary, or other organ involvement.

**Table 1:** Abnormal laboratory investigations.

Test Item	Test Result	Reference Range	Unit
LYMPH%	17.9	20–50	–
MONO%	11.8	3.0–10	–
CRP	12.48	0–3	mg/L

### Clinical course

**Figure 2:**
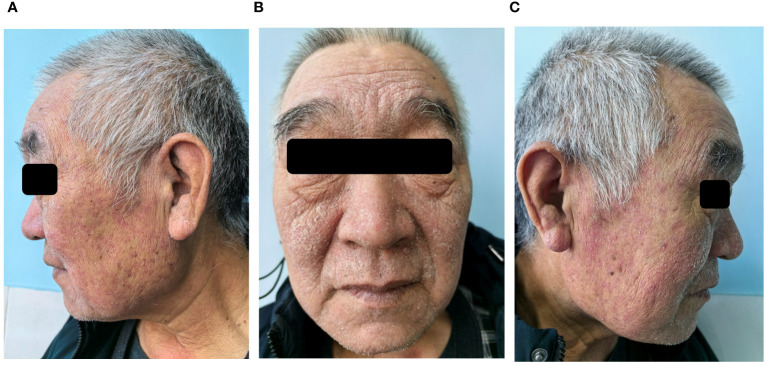
After seven days of follow-up after discharge, seborrheic dermatitis had worsened. Patches of greasy skin covered with flaky white or yellow scales on face, sides of the nose, and eyebrows **(A-C)**.

Based on the medical history, clinical presentations and laboratory data, the patient was diagnosed with efgartigimod-associated KVE and herpetic conjunctivitis. After 2 days of antiviral therapy with intravenous acyclovir (5mg/kg, 3 times a day, every 8 hours), the patient’s body temperature returned to normal. Scab formation at the site of vesicles and facial edema subsided 4 days later. The ophthalmic manifestations gradually alleviated too. After completing 10 days of antiviral therapy, the patient was cured and his scabs healed, leaving a red mark without any other residual complications. However, the patient returned to our dermatology clinic 7 days later, his pre-existing skin disease, seborrheic dermatitis, had worsened due to skin barrier dysfunction caused by KVE. There were patches of greasy skin covered with flaky white or yellow scales on his face, sides of the nose, and eyebrows (**Figure 2**). Malassezia was found in skin scrapings under a microscope. The patient was prescribed 1% terbinafine hydrochloride cream and barrier repair lotion. After more than 20 days, the patient’s seborrheic dermatitis gradually improved. After 4 months of follow-up, the patient did not experience HSV reactivation, and his symptoms of OMG have improved compared to before.

## Discussion

Efgartigimod is a novel FcRn antagonist that can reduce circulating IgG levels, including pathogenic IgG, and is a drug used to treat IgG-mediated autoimmune diseases. Efgartigimod was approved by the FDA in December 2021 for the treatment of AChR Ab+ MG patients. There is currently no publicly available randomized study on the treatment of AChR Ab+ OMG with efgartigimod. We look forward to more clinical trials in the future. Intravenous efgartigimod was generally well tolerated in patients with MG during the ADAPT study (3). As efgartigimod causes a transient decrease in IgG levels, immunization with live or live-attenuated vaccines is not recommended during treatment (4). The levels of protective antibodies against tetanus toxoid, varicella zoster virus, and pneumococcal capsular polysaccharide decreased along with serum IgG levels with efgartigimod treatment in pemphigus (5). The protective antibodies returned to baseline levels upon cessation of treatment.

KVE, also called eczema herpeticum, refers to a disseminated skin infection due to a virus that usually leads to localized vesicular eruptions, on the basis of eczema, atopic dermatitis, or other cutaneous disease (6). KVE may be associated with systemic symptoms such as high temperature, malaise, and swollen lymph nodes near the infection. HSV is considered the main causative agent. A defective skin barrier combined with immune deficiencies seems to lead to infection with HSV, and both cell-mediated and humoral immunity dysfunction are implicated. The Tzanck smear, viral cultures, histology or detection of viral DNA by polymerase chain reaction (PCR), and electron microscopy may be helpful in doubtful cases. It is a rare but potentially life-threatening disorder. Rare complications of HSV include meningoencephalitis and disseminated infection or keratitis. M. Okamoto et al. reported a case of KVE progressing to HSV hepatitis in an immunocompetent patient (7). Antiviral therapy is effective, but it should be started as soon as possible after diagnosis to reduce the incidence rate and mortality. Orally administer 400–800 mg of acyclovir 5 times a day, or 1 g of valaciclovir 2 times a day for 10–14 days or until the lesion heals. Intravenous acyclovir (5–10mg/kg, 3 times a day, every 8 hours) is prescribed if the patient is too sick to take tablets, or if the infection is deteriorating despite treatment.

Considering that the first-line treatment was ineffective for AChR Ab+ OMG in our patient, his attending neurologist suggested switching to intravenous efgartigimod. KVE and herpetic conjunctivitis occurred 3 days after one treatment cycle of intravenous efgartigimod. During the 8 years following intermittent immunosuppressive therapy, the patient never experienced HSV reactivation. Therefore, we suspected that efgartigimod played a major role in this unexpected infection. The mechanisms underlying the pathogenesis of viral reactivation may be that the levels of protective antibodies against HSV decreased to below the threshold along with serum IgG levels with efgartigimod treatment. But whether the levels of antibody against HSV are strongly correlated with disease severity or reactivation from latency has not been well established (8). This case undoubtedly supports the protective effect of comprehensive antibodies. The necessity of monitoring the serum IgG levels of patients in subsequent treatment still requires clinical observation with a large sample size. Of course, the skin barrier dysfunction caused by seborrheic dermatitis on the patient’s face provided an opportunity for HSV. Patients with MG may also have other dermatosis with skin barrier dysfunction, which should be well cared for before starting the FcRn antagonist. Therefore, during FcRn antagonist therapy, when patients have skin barrier dysfunction and low levels of HSV IgG, the risk of virus reactivation is high. It is recommended to use systemic antiviral drugs for prevention.

Efgartigimod exhibited an early effect on disease activity and outcome parameters as a therapy for pemphigus in a phase 2 clinical trial (9). T. Demitsu et al. reported a fatal case of recalcitrant pemphigus foliaceus with KVE eruption (10). Severely painful skin eruptions, erosions, and ulcers have been identified as important initial signs of a HSV infection. When the erosions or ulcers are accompanied by a bacterial infection, such as pseudomonas aeruginosa, the clinical diagnosis can be more confusing (11). It is also necessary to comprehensively evaluate whether there is any systematic involvement. Thus, multidisciplinary consultation becomes particularly important.

## Conclusion

Efgartigimod is a well-tolerated drug with minor side effects. To date, no cases of efgartigimod-associated KVE and herpetic conjunctivitis in patients with AChR Ab+ OMG has been reported in literature. Clinicians should recognize the rare infection especially when patients have the skin barrier dysfunction. Antiviral therapy should be started as soon as possible after diagnosis.

## Data availability statement

The original contributions presented in the study are included in the article/supplementary material. Further inquiries can be directed to the corresponding authors.

## Ethics statement

The studies involving humans were approved by Ethics Committee of the Second Affiliated Hospital of Shandong First Medical University. The studies were conducted in accordance with the local legislation and institutional requirements. The participants provided their written informed consent to participate in this study. Written informed consent was obtained from the individual(s) for the publication of any potentially identifiable images or data included in this article.

## Author contributions

LG: Writing – original draft, Writing – review & editing, Data curation, Conceptualization. YL: Methodology, Writing – original draft. YS: Writing – review & editing, Formal analysis. WC: Writing – review & editing, Formal analysis. XN: Writing – review & editing, Formal analysis. FW: Project administration, Writing – original draft, Supervision. ZM: Project administration, Writing – original draft.
